# Type 2 Deiodinase Promotes Fatty Adipogenesis in Muscle Fibroadipogenic Progenitors From Adult Male Mice

**DOI:** 10.1210/endocr/bqaf050

**Published:** 2025-03-10

**Authors:** Cristina Luongo, Daniela Di Girolamo, Raffaele Ambrosio, Sara Di Cintio, Maria Angela De Stefano, Tommaso Porcelli, Domenico Salvatore

**Affiliations:** Department of Clinical Medicine and Surgery, University of Naples Federico II, Naples 80131, Italy; Department of Biology, University of Naples Federico II, Naples 80131, Italy; CEINGE Biotecnologie Avanzate Franco Salvatore, Naples 80131, Italy; Department of Public Health, University of Naples Federico II, Naples 80131, Italy; Department of Public Health, University of Naples Federico II, Naples 80131, Italy; Department of Public Health, University of Naples Federico II, Naples 80131, Italy; Department of Public Health, University of Naples Federico II, Naples 80131, Italy; CEINGE Biotecnologie Avanzate Franco Salvatore, Naples 80131, Italy; Department of Public Health, University of Naples Federico II, Naples 80131, Italy

**Keywords:** deiodinase, thyroid hormone, adipogenesis, fibro-adipogenic progenitors

## Abstract

Fibro-adipogenic progenitor cells (FAPs) are a heterogeneous population of multipotent mesenchymal cells that give rise to fibroblasts and adipocytes. In response to muscle injury, FAPs are activated and cooperate with inflammatory and muscle stem cells to promote muscle regeneration. In pathological conditions, such as muscular dystrophies, this coordinated response is partially lost and an accumulation of FAPs is observed that is responsible for maladaptive fibrosis, ectopic fat deposition, and impaired muscle regeneration. The role of intracellular thyroid hormone (TH) signaling in this cellular context is largely unknown. Here we show that intracellular 3,5,3′-triiodothyronine (T3) concentration in FAPs is increased in vitro during adipogenic differentiation via the increase of the T3-producing type 2 deiodinase (D2). The adipogenic potential is reduced in FAPs cultured in the presence of 3,3,5′-triiodothyronine (rT3), a specific D2 inhibitor, while exogenous administration of THs is able to induce the expression of relevant adipogenic genes. Accordingly, on genetic D2 depletion in vivo, adipogenesis was significantly reduced in D2KO compared to control mice. These data were confirmed using a FAP-inducible specific D2-KO mouse model, suggesting that a cell-specific D2-depletion in FAPs is sufficient to decrease fatty muscle infiltration and to improve muscle regeneration. Taken together, these data show that TH signaling is dynamically modulated in FAPs wherein D2-produced T3 is required to promote maturation of FAPs into adipocytes.

The thyroid hormone (TH) intracellular concentration is regulated by the dynamic expression of a family of enzymes named deiodinases. Two iodothyronine deiodinase enzymes (D1 and D2) catalyze the deiodination of thyroxine (T4) into T3, the most biologically active form of TH. The third member of the deiodinase family, D3, has an opposite function, catalyzing the inactivation of both T4 and T3 (1). Thus, deiodinase enzymes tightly control the intracellular concentration of TH at the cellular level, and provide a potent mechanism for prereceptor regulation of TH action.

Adipogenesis is a multistep process consisting of two phases: proliferation of preadipocytes and their differentiation into mature adipocytes. This process is regulated by several transcription factors, many of which are TH target genes (2). In fetal brown adipose tissue, type 2 deiodinase (D2), the enzyme converting T4 into T3, is known to be upregulated during this process (2).

Intramuscular fatty cell infiltration is a characteristic feature of several pathological conditions such as sarcopenia, diabetes, cachexia, and muscular dystrophies, all of which adversely affect muscle function (3, 4). Increased intramuscular adipose tissue deposition is strongly associated with reduced force production and overall muscle function. In muscular dystrophies, intramuscular fatty infiltration positively correlates with disease severity (5). The mechanisms regulating muscle fatty infiltration are still not fully understood. Some studies have shown that fibro-adipogenic progenitor cells (FAPs), an interstitial progenitor of mesenchymal origin, can give rise to adipocytes and myofibroblasts (6) and are responsible for fatty degeneration and the fibrosis following chronic muscle injury.

FAPs have also been implicated in muscle homeostasis and regeneration following acute injury, wherein they participate in the activation and expansion of muscle stem cells (MuSCs) by secreting paracrine factor (7-9). Under healthy conditions, FAPs provide a supportive environment for myogenic cells, producing extracellular matrix components, cytokines, and growth factors that regulate myogenesis and muscle growth (8). Depletion of FAPs from mouse muscle, under homeostatic conditions, induces muscle atrophy and loss of MuSCs, demonstrating that FAPs are required for the maintenance both of skeletal muscle integrity and muscle stem cell pool (10). On acute muscle injury, immune cells activate FAPs, which begin to proliferate (11, 12). Following activation and expansion of MuSCs, FAPs are removed from the injured site by apoptosis, while activated MuSCs continue to undergo myogenesis (6). In pathological conditions, such as muscle dystrophies, FAPs activities are disorganized; they continue to proliferate and finally differentiate into adipocytes and fibroblasts. We have recently demonstrated that D2 and D3 are expressed in FAPs, which implicates a role for these enzymes in this cell context (13).

In this paper, we analyzed the role of intracellular TH action in driving adipocyte-fibroblast fate decisions in FAPs from adult muscle. We identified a cell-autonomous mechanism by which FAP adipogenic differentiation required an increase in thyroid status that was achieved, at least in part, by the increased expression of D2. We also showed that adipogenic differentiation is significantly reduced both in a global *Dio2* knockout (KO) mouse model (D2KO) and FAP-specific conditional *Dio2* depletion (FAP-D2KO) mouse models after in vivo induction of muscle fatty infiltration by glycerol injection.

## Materials and Methods

### Animals

Dio2fl/fl (14), global-D2KO (15), C57BL/6, and B6N.Cg-Tg (Pdgfra-cre/ERT)467Dbe/J (the last two were purchased from Charles River Stock No. 027 and 18280, respectively) mice were used in this study. Mice were housed in a pathogen-free facility at CEINGE Biotecnologie Avanzate, Naples, Italy. Experiments were performed according to the guidelines of the Ministero della Salute and approved by the Institutional Animal Care and Use Committee (IACUC: 63/2023-PR). Housing conditions included temperature approximately 72 °F (∼22 °C), 40% to 60% humidity, 14/10 light/dark daily cycle. Only male mice aged 8 to 12 weeks were used for the experiments. For the muscle injury, mice were anesthetized by a ketamine (90 mg/kg)-xylazine (10 mg/kg) cocktail and 25 μL of 50% Glycerol (Sigma-Aldrich, G9012) in saline solution were injected into and along the length of the right tibialis anterior (TA) and 50 μL of 50% glycerol in saline solution were injected into and along the length of the right gastrocnemius (GC). We used uninjected contralateral muscles as control.

Tamoxifen (Sigma Aldrich, T5648) was dissolved in corn oil (Sigma Aldrich, C8267)/10% ethanol (Carlo Erba, No. 4146052) at a concentration of 10 mg/mL and stored at 4 °C. Mice were injected intraperitoneally with tamoxifen at 80 mg/kg of body weight for experiments involving inducible CreERT. In the experiments, both genotypes (Pdgfrα-CreERT2; Dio2fl/fl [FAP-D2KO] and Dio2fl/fl [FAP-D2WT]) received tamoxifen treatment.

### Cell Preparation and Fluorescence-Activated Cell Sorter Isolation

To isolate FAPS, adult limb muscles were dissected, minced, and incubated with a mix of Dispase II (Roche, 04942078001) 3 U/mL and Collagenase A (Roche, 11088793001) 1 mg/mL in phosphate-buffered saline 1× at 37 °C in a water bath for 1 hour. The muscle suspension was successively filtered through 100-μm and 70-μm cell strainers and then spun at 50*g* for 10 minutes at 4 °C to remove large tissue fragments. The supernatant was collected and washed twice by centrifugation at 600*g* for 15 minutes. Cells were stained with antibodies Pacific Blue SCA-1 (108120 Biolegend), APC CD31 (551262, BD Biosciences), APC CD45 (559864, BD Biosciences), and APC a7 integrin (FAB3518A, R&D System) for 30 minutes on ice. All antibodies used in this paper are listed in Supplementary Table S1 (16). Cells were finally washed and with cold Dulbecco’s modified Eagle’s medium (DMEM) and then resuspended in DMED 2% fetal bovine serum (FBS) for sorting using FACSAria III, BS Biosciences. Fibroadipogenic progenitors were sorted based on positive staining for Sca1 and absence of staining for α 7-integrin, CD31, and CD45.

In experiments for which FAPs and MuSCs were isolated from the same muscle, we used the MACS Cell Separation System for sorting. First, the muscle suspension was filtered through 70-μm cell strainers. MuSCs were then isolated using the Satellite Cell Isolation Kit (Miltenyi Biotech, 130-098-463) according to the manufacturer's instructions. From the MuSC-negative cell fraction, we further isolated PDGFRα-positive cells using the CD140a (PDGFRα) MicroBead Kit (Miltenyi Biotech, 130-101-502). Within this population, we then selected Sca1 + cells using the Anti-Sca-1 (non-HSC) MicroBeads (Miltenyi Biotech, 130-106-641), corresponding to FAPs.

### Cell Culture, Transfection, and Infection

FAPs were grown in high-glucose DMEM (D6546, Sigma) containing 20% FBS (Invitrogen) and 1% penicillin/streptomycin (P/S, Invitrogen). For adipogenic differentiation, after 4 days of non adipogenic induction medium (NI), cells were exposed for 4 days to adipogenic induction medium consisting of DMEM with 10% FBS (D6546, Sigma), 0.25 mM indomethacin (I7378, Sigma), 0.5 mM IBMX (28822-58-4, Sigma), 1 μM dexamethasone (D4902, Sigma), and 10 μg/mL insulin (I2642, Sigma), followed by a further 3 days in adipogenic maintenance medium, consisting of DMEM with 10% FBS and 10 μg/mL insulin (I2642, Sigma). Where indicated, TH (30 nM T3 plus 30 nM T4) or rT3 (30 nM) was added to the adipogenic induction and differentiation media. All cells were cultured in a 5% CO_2_ atmosphere at 37 °C. FAPs were transfected with lipofectamin 2000 (Life Technologies) according to the manufacturer's instructions. Adenoviruses encoding CRE recombinase (FAP-Dio2–depleted cells) or green fluorescent protein (GFP) (FAP control cells) were added to FAPs at a multiplicity of infection of 250 and incubated for 2 hours at 37 °C in medium serum free 24 hours before the induction of adipogenic differentiation when cell confluence was around 70%. Cells were collected 7 days post infection.

### Luciferase Assay

Luc plasmid TRE3-TK-Luc and CMV-*Renilla* reporters were cotransfected into FAP cells and induced to differentiate as indicated. Luciferase activities were measured 48 hours after transfection using the Dual Luciferase Reporter Assay System (Promega Corp), and differences in transfection efficiency were corrected relative to the level of *Renilla* activity. Each construct was studied in triplicate in at least 3 separate transfection experiments.

### Western Blot Analysis

Total protein extracts from FAPs or from injured muscle were run on a 10% sodium dodecyl sulfate–polyacrylamide gel electrophoresis gel and transferred onto an Immobilon-P transfer membrane (Millipore). The membrane was then blocked with 5% nonfat dry milk in PBS, probed with anti-perilipin (1:1000, ab3526; Abcam), anti–peroxisome proliferator-activated receptor (PPAR)-γ antibody (1:1000, E-AB-32647; Elabscience), and anti-PRDM16 (1:1000, ab106410; Abcam) for 2 hours, washed and incubated with horseradish peroxidase–conjugated donkey anti-rabbit immunoglobulin G (IgG) secondary antibody (1:3000), and detected by chemiluminescence (Millipore, WBKLS0500). After extensive washing, the membrane was incubated with anti–GAPDH-specific antibodies (1:5000, E-AB-20059; Elabscience.) as loading control. All antibodies used in this work are listed in Table 1.

**Table 1.** Antibodies list

Name	RRID
Pacific Blue anti-mouse Ly-6A/E (Sca-1)	AB_493273
Rat Anti-CD31 Monoclonal Antibody, Allophycocyanin Conjugated, Clone MEC 13.3	AB_398497
Rat Anti-CD45 Monoclonal Antibody, Allophycocyanin Conjugated, Clone 30-F11	AB_398672
Mouse Integrin α 7 APC-conjugated antibody	AB_1026275
Perilipin A antibody	AB_2167274
Anti–PPAR-γ antibody produced in rabbit	AB_10745651
Goat anti-Rabbit IgG (H + L)-HRP Conjugate	AB_11125142
Goat anti-Rabbit IgG (H + L) Cross-Adsorbed Secondary Antibody, Alexa Fluor 594	AB_2534079
Goat anti-Rabbit IgG (H + L) Cross-Adsorbed Secondary Antibody, Alexa Fluor 488	AB_143165

Abbreviations: HRP, horseradish peroxidase; IgG, immunoglobulin G; PPAR, peroxisome proliferator-activated receptor.

### Immunofluorescence and Histology

For immunofluorescent staining, cells were fixed with 4% formaldehyde and permeabilized in 0.1% Triton X-100, then blocked with 20% goat serum and incubated with primary antibody. Dissected muscle was snap-frozen in liquid nitrogen, sectioned (7 μm thick), and stained with hematoxylin and eosin stain (Sigma, GHS116 Hematoxylin, HT110216 Eosin), Oil Red (Sigma, O0625), Sirius red (Sigma, 365548), or immunofluorescence using standard protocols. Alexa 594 or 488-conjugated secondary antibody was used (Life Technologies). Images were acquired with an IX51 Olympus microscope and Cell∗F software. Images were assembled using Adobe Photoshop.

### Real-Time Quantitative Reverse Transcription–Polymerase Chain Reaction

Messenger RNAs (mRNAs) were extracted with TRIzol reagent (Life Technologies). Complementary DNAs (cDNAs) were prepared with SuperScript VILO Master Mix (Life Technologies) as indicated by the manufacturer. The cDNAs were amplified by polymerase chain reaction (PCR) in an iQ5 Multicolor Real-Time Detector System (Bio-Rad) with the fluorescent double-stranded DNA-binding dye SYBR Green (Applied Biosystems). Specific primers for each gene were designed to work under the same cycling conditions (95 °C for 10 minutes followed by 40 cycles at 95 °C for 15 seconds and 60 °C for 1 minute), generating products of comparable sizes (∼200 bp for each amplification). Primer combinations were positioned whenever possible to span an exon-exon junction and the RNA digested with deoxyribonuclease to avoid genomic DNA interference. For each reaction, standard curves for reference genes were constructed based on six 4-fold serial dilutions of cDNA. All samples were run in triplicate. The template concentration was calculated from the cycle number when the amount of PCR product passed a threshold established in the exponential phase of the PCR. The relative amounts of gene expression were calculated with cyclophilin A expression as an internal standard (calibrator). The results, expressed as n-fold differences in target gene expression, were determined as follows: n × target = 2^(ΔCtsample – ΔCtcalibrator)^.

### Statistical Analysis

Statistical analysis was calculated using one-way analysis of variance (≥3 groups to compare) or Mann-Whitney (2 groups to compare) tests and a *P* value less than .05 was assumed as statistical significance (*P < .05; **P < .01; ***P < .001). In the figures, error bars represent SD. The statistical analysis, reported in the figures, represents the Tukey multiple comparison test result relative to comparison of the two groups indicated in each corresponding graph. Statistical analysis was performed using the GraphPad software 9.

## Results

### Deiodinases (*Dio*s) Expression Is Modulated in Fibro-Adipogenic Progenitor Cells During Adipogenic Differentiation

**Figure 1.**
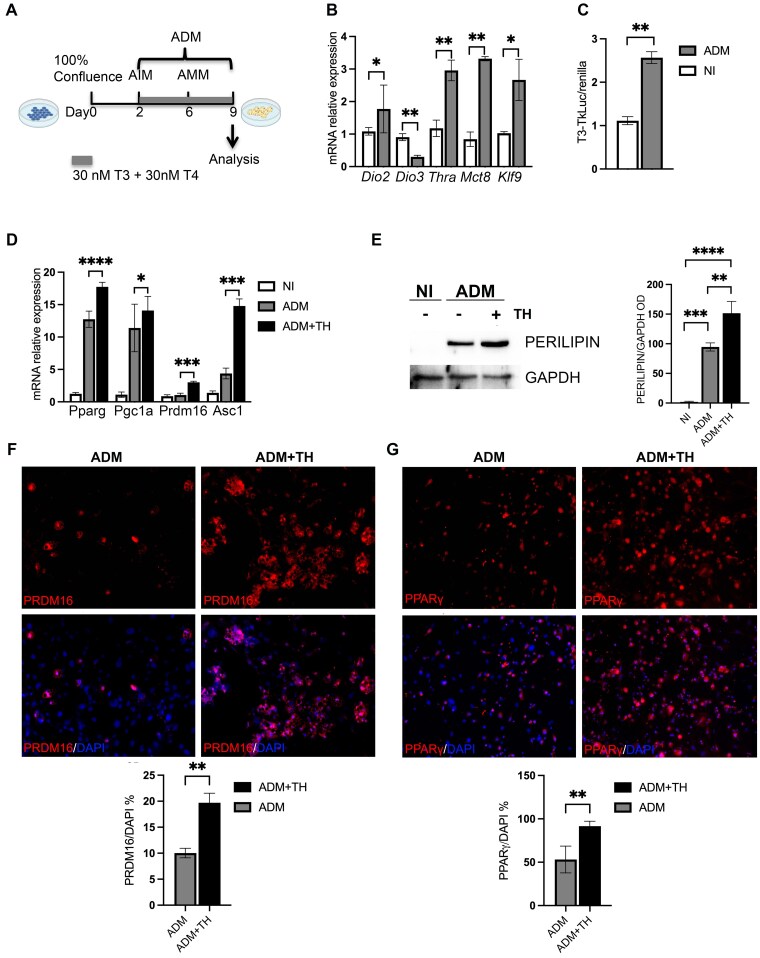
A, Thyroid hormone (TH) status increases during a fibro-adipogenic progenitor cell (FAP) adipogenic differentiation in vitro experimental regimen. Adult FAPs isolated from C57BL/6 were plated in growth media. After 2 days cells were treated with Adipogenic Induction Medium (AIM) for another 4 days and then the cells were kept in Adipogenic Maintenance Medium (AMM) to induce final adipogenic differentiation (AIM + AMM = ADM). B, Real-time quantitative polymerase chain reaction (qPCR) on FAPs cultured under adipogenic vs proliferative conditions for *Dio2*, *Dio3*, *Trha, Mct10*, and *Klf9*. *Gapdh* was used as the housekeeping gene. C, Luciferase assay of FAPs cells transiently transfected with the TRE3-TK-LUC promoter and cultured under proliferative or adipogenic conditions. The results are shown as means of the LUC/Renilla ratio. D, Real-time qPCR on FAPs cultured under proliferative condition (white), or adipogenic condition in the presence of or absence of TH for adipogenic genes in. *Gapdh* was used as the housekeeping gene. E, Western blot on FAPs cultured under proliferative or adipogenic conditions in the presence of or absence of TH showing the expression of perilipin. GAPDH is used as the loading control. The bar plot reports the densitometric values of perilipin in the 3 conditions normalized on the densitometric values of GAPDH. F and G, Immunofluorescence analysis for PRDM16 (in F) and PPARγ (in G) in differentiated FAPs after exposure to TH (30 nM) or vehicle. Nuclei were revealed using DAPI (4′,6-diamidino-2-phenylindole). The bar plots on the right report the percentage of PRDM16 PPARγ-positive cells in both conditions.

Recently, we detected high levels of deiodinases (D2 and D3), TH transporters (THT), and receptors (TR) (13) mRNAs in FAPs isolated from mouse hindlimb muscle, which suggests active TH signaling in this cell context. Given the ability of FAPs to differentiate into different cell types, including fibroblasts and adipocytes, we hypothesized that intracellular regulation of TH concentrations via deiodinases might play a role in FAP cell fate decision. To address this point, freshly fluorescence-activated cell sorter (FACS)-isolated FAPs from mouse hind limbs were cultured under proliferative or adipogenic differentiation conditions as described in “Materials and Methods” (Fig. 1A). Interestingly, on adipocyte differentiation, *Dio2*, THR-α (*Thra*), and THT *MCT8* mRNA expression was upregulated compared to proliferative conditions (NI); conversely and functionally consistent, *Dio3* mRNA expression was downregulated (Fig. 1B). These gene modifications were indicative of an increased TH signaling, as suggested also by the increased expression of *Klf9*, a well-known TH-responsive gene (see Fig. 1B) (10.1182/blood-2017-05-783043). To prove this, we measured T3 transcriptional activity by using as a proxy the artificial TRE3-TKLuc promoter (carrying 3 TRE binding sites upstream of the Luciferase gene). T3 signaling was significantly induced during adipogenic differentiation (ADM) compared to proliferative conditions (NI) (Fig. 1C).

Overall, these results indicated that along with the increase in *Dio2* levels, increased TH signaling is associated with FAPs adipogenic differentiation *in vitro*.

### Thyroid Hormone Enhances the In Vitro Adipogenic Potential of Fibro-Adipogenic Progenitor Cells

To assess whether exogenously added TH (compared to TH normally present in the culture media) can potentiate adipogenesis, we cultured freshly isolated FAPs in the presence or absence of supraphysiological TH levels during in vitro adipogenic differentiation. We found that TH treatment increases the expression of relevant adipogenic markers including *Pparg*, *Prdm16*, *Pgc1a*, and *Asc1* (Fig. 1D). Accordingly, Western blot for perilipin, a marker of mature adipocytes, was increased by TH treatment (Fig. 1E). Finally, immunofluorescence analysis showed that PRDM16 and PPARγ protein levels were significantly higher in FAPs undergoing adipogenic differentiation in the presence of TH (Fig. 1F and 1G).

### Type 2 Deiodinase–derived 3,5,3′-Triiodothyronine Is Required for the Adipogenic Potential of Fibro-Adipogenic Progenitor Cells

**Figure 2.**
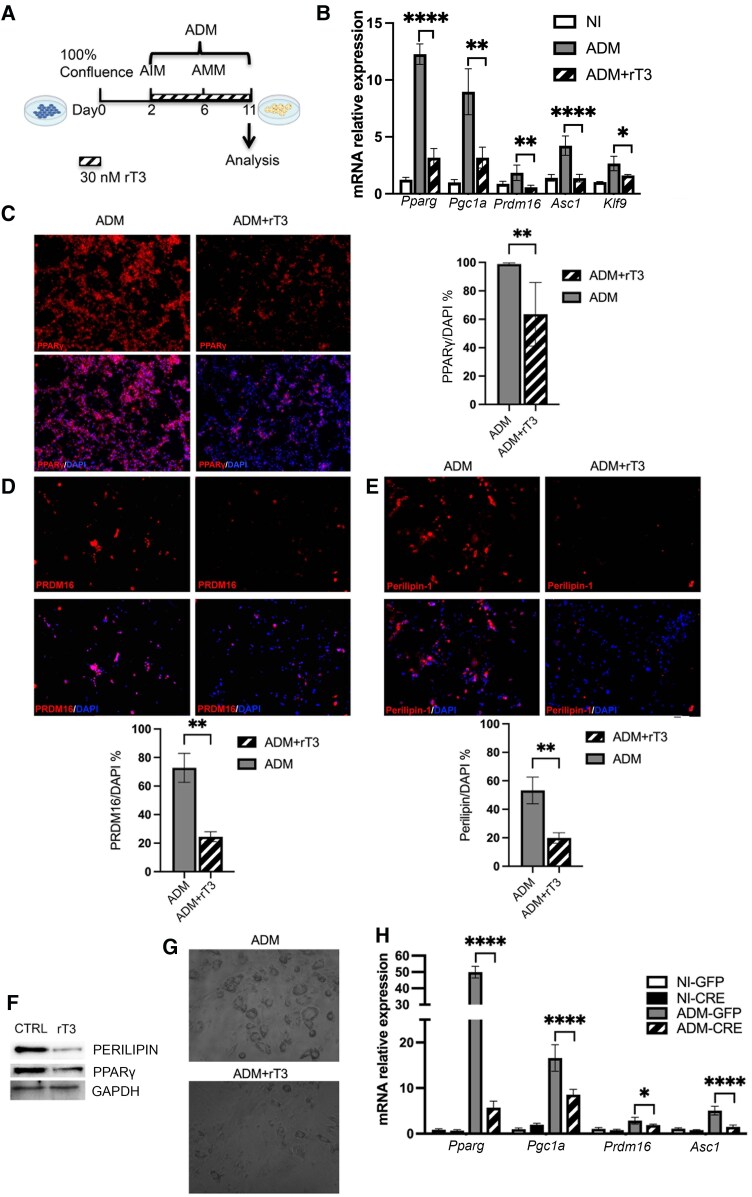
The inhibition of type 2 deiodinase (D2) impairs fibro-adipogenic progenitor cell (FAP) adipogenic differentiation. A, Experimental regimen. Adult FAPs isolated from C57BL/6 were plated in growth media. After 2 days cells were treated with Adipogenic Induction Medium (AIM) for 4 days, and then the cells were kept in Adipogenic Maintenance Medium (AMM) to induce final adipogenic differentiation (ADM). During the ADM treatment cells were cultured in the presence or absence of 30 nM 3,3,5′-triiodothyronine (rT3). This figure was created with BioRender. B, Real-time quantitative polymerase chain reaction (qPCR) showing the messenger RNA (mRNA) expression levels of indicated adipogenic genes and *Klf9* on FAPs cultured under proliferative condition, or adipogenic condition in the presence of or absence of rT3. *Gapdh* was used as the housekeeping gene. C to E, Immunofluorescence analysis for C, PPARγ; D, PRDM16; and E, Perilipin and in differentiated FAPs after the exposure to rT3 (30 nM) or vehicle. Nuclei were revealed using DAPI (4′,6-diamidino-2-phenylindole). The bar plots below the images report the percentage of Perilipin-PRDM16 and PPARγ-positive cells. F, Western blot shows the expression of perilipin and PPARγ protein in FAPs cultured under adipogenic condition in the presence or absence of rT3. GAPDH is used as the loading control. G, Morphology of FAPs under adipogenic condition in the absence (upper) and absence (lower) of rT3. H, Real-time qPCR of transduced adult FAPs with green fluorescent protein (GFP)-control or CRE for adipogenic markers under proliferative and adipogenic conditions. *Gapdh* was used as the housekeeping gene.

We hypothesized that upregulation of *Dio2* in FAPs was necessary to achieve fully adipogenic differentiation. To test this hypothesis, we analyzed FAP adipogenic potential in the absence of D2. First, FAPs were cultured under adipogenic condition in the presence or absence of rT3, a specific D2 inhibitor (17) (Fig. 2A). The rT3 treatment decrease the intracellular TH status as suggested by the reduction of the expression of *Klf9* (Fig. 2B). Interestingly, rT3 treatment significantly attenuates adipogenic differentiation of FAPs in vitro, as demonstrated by the blunted raise in adipogenic markers such as *Pparg*, *Prdm16*, *Pgc1a*, and *Asc1* (see Fig. 2B). Moreover, immunofluorescence analysis showed that PPARγ, perilipin, and PRDM16 protein levels were significantly reduced in FAPs undergoing adipogenic differentiation in the presence of rT3 (Fig. 2C-2E). These data were also confirmed by Western blot analysis (Fig. 2F) and by the morphology of the treated cells, which showed much higher lipid content when D2 is functional (Fig. 2G).

To further validate these findings, we genetically depleted D2 in FAPS. To do this, FAPs from *dio2^fl/fl^* mice were infected in vitro with Adeno-Cre (Ad-CRE) virus, or Adeno-GFP (Ad-GFP) virus as control under proliferative (NI) and adipogenic conditions (ADM). Consistent with what was observed with D2 enzymatic inhibition, genetic *Dio2* ablation significantly reduces FAPs adipogenic differentiation, as demonstrated by the reduced levels of *Prdm16*, *Pgc1a*, *Pparγ*, and *Asc1* in Dio2^−/−^ FAPs (Fig. 2H).

### In Vivo Genetic Type 2 Deiodinase–depletion Reduced Glycerol-Induced Fatty Infiltration

**Figure 3.**
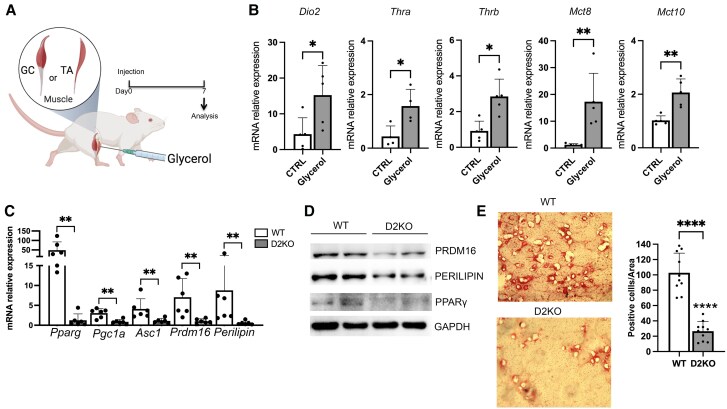
In vivo genetic deletion of *Dio*2 decreases intramuscular fatty infiltration. A, Experimental regimen. Two-month-old mice were injected with 50% glycerol or phosphate-buffered saline as control. Muscles were collected 7 days post injection. This figure was created with BioRender. B, Real-time quantitative polymerase chain reaction (qPCR) showing the messenger RNA (mRNA) expression levels of *Dio2*, *Trha*, *Trhb*, *Mct8*, and *Mct10* genes in glycerol-injected muscle compared to resting muscle. *Gapdh* was used as the housekeeping gene (control mice n = 4, D2KO mice n = 5). C, Real-time qPCR showing the mRNA expression levels of indicated adipogenic genes in gastrocnemius muscles from wild-type (WT) and D2KO mice 7 days post glycerol injection (control mice n = 6, D2KO mice n = 6). *Gapdh* was used as the housekeeping gene. The noninjured WT muscle has been used as control (not showed in the graph). D, Western blot showing the expression of PRDM16, perilipin, and PPARγ protein in gastrocnemius muscle from WT and D2KO mice 7 days post glycerol injection. GAPDH was used as the loading control. E, Red Oil staining on muscle from WT and D2KO mice 7 days post glycerol injection. The associated bar plot reports the percentage of Red Oil–positive cells.

To verify whether D2 is involved in the process of FAP adipogenic differentiation in vivo, we took advantage of the glycerol muscle injury model, which has been described as an in vivo model to study muscle fatty infiltration (18). We injected glycerol into muscles of C57BL/6 mice (25 µL into TA and 50 µL into GC) to induce adipogenesis and, 7 days later, we measured deiodinase, THT, and TR mRNA expression in injected vs controlateral muscles (used as control muscle) (Fig. 3A). Consistent with the in vitro results, we found *Dio2*, major THT, and TR expression was strongly upregulated during adipogenesis in vivo (Fig. 3B).

To assess the functional role played by D2 during adipogenic differentiation in vivo, we induced muscle fatty infiltration in D2KO mice. Seven days after glycerol injection, we observed that in vivo adipogenesis was impaired in D2KO, as demonstrated by the reduced *Pparg*, *Pgc1a*, *Asc1*, *Prdm16*, and *perilipin* mRNA expression levels (Fig. 3C). To better evaluate the inhibitory effect of D2 depletion on fat deposition, we measured protein levels of some adipogenic markers and we found that PPARγ, PRDM16, and perilipin protein levels were reduced in D2KO mice compared to wild-type (WT) mice (Fig. 3D). Of note, Oil Red O staining performed on muscle sections after 7 days on glycerol injection confirmed a decreased number of Red Oil+ cells (differentiated adipocytes) in D2KO vs WT mice (Fig. 3E).

To assess whether the defective adipogenesis in global D2KO was directly related to the D2-deletion specifically in adipocyte progenitors, we generated a FAP-specific conditional D2 KO mouse by crossing a *Pdgfrα-CreER^T2^* mouse with a *Dio2*^fl/fl^ mouse (named FAP-D2KO). FAP-D2KO mice are viable and have no substantial differences in body weight, growth, litter size, or reproductive capacity in either sex when compared with D2^fl/fl^ littermates (Table 2, Supplementary Fig. S1A (16)). Dio2 mRNA levels were reduced by approximately 50% in FAP-D2KO mice compared to controls, confirming considerable recombination of the *Dio2* gene (data not shown). However, no differences in Dio2 mRNA expression were observed in MuSCs isolated from the resting hindlimb muscle of FAP-D2WT and FAP-D2KO mice (Supplementary Fig. S1B (16)). Likewise, *Dio2* mRNA expression remained unchanged in tissues with elevated Dio2 expression, such as brown adipose tissue, in both genotypes (Supplementary Fig. S1C (16)). Furthermore, *Dio2* deletion in FAPs did not affect the expression of *Dio3*, *Thra*, *Thrb*, or *Mct8* under resting conditions (data not shown).

**Table 2.** Characteristics littermate *Pdgfrα-CreERT2;Dio2fl/fl* (FAP-D2KO)

Littermate		*P*
No. of littermate	21	NA
No. of mice	151	NA
Littermate size, average	7	NA
Sex		
Male	76 (50%)	>.9999
Female	75 (50%)	
Cre frequency		
Male		>.9999
CRE+	36 (47.4%)	
CRE–	40 (52.6%)	
Female		
CRE+	36 (48%)	
CRE–	39 (52%)	

**Figure 4.**
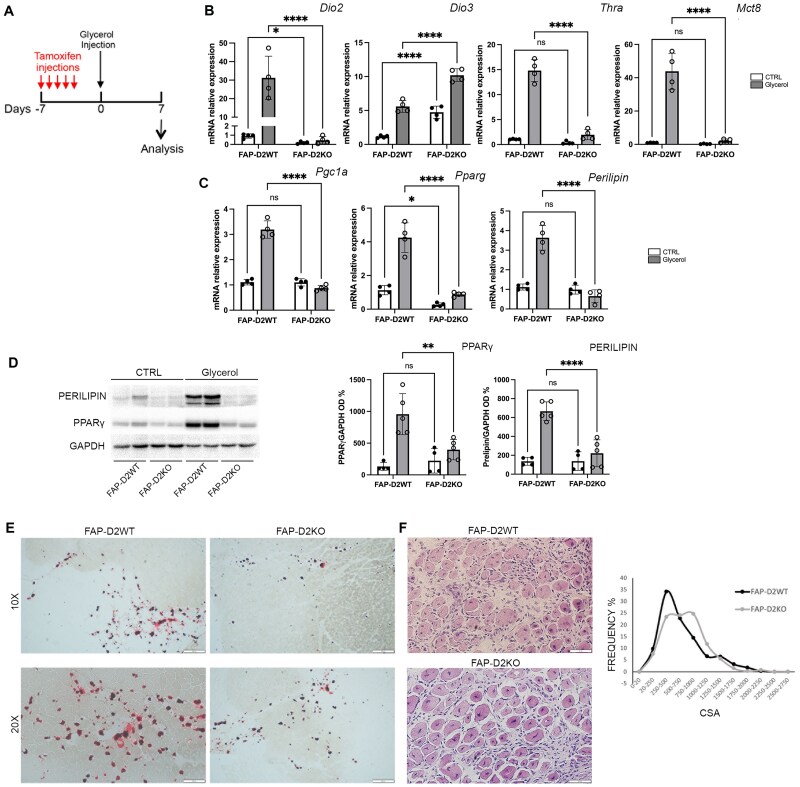
In vivo genetic ablation of *Dio*2 from fibro-adipogenic progenitor cells (FAPs) decreases intramuscular fatty infiltration. A, Experimental regimen. Two-month-old FAP-D2WT and FAP-D2KO mice were injected with tamoxifen for 5 days. After 2 days, 50% glycerol or phosphate-buffered saline as control were injected in tibialis anterior (TA) or gastrocnemius (GC) muscle. Muscles were collected 7 days post glycerol injection and FAP cells were isolated by MACS microbeads. This figure was created with BioRender. B, Real-time quantitative polymerase chain reaction (qPCR) showing the messenger RNA (mRNA) expression levels of *Dio2*, *Dio3*, *Trha*, and *Mct8* in FAPs isolated from FAP-D2WT (n = 4) and FAP-D2KO (n = 4) glycerol-injected muscle. *Gapdh* was used as the housekeeping gene. C, Real-time qPCR showing the mRNA expression levels of indicated adipogenic genes in FAPs isolated from FAP-D2WT (n = 4) and FAP-D2KO (n = 4) glycerol-injected muscle. *Gapdh* was used as the housekeeping gene. D, Western blot showing the expression of perilipin and PPARγ protein in GC muscle from FAP-D2WT and FAP-D2KO mice 7 days after the injection of glycerol. GAPDH was used as the loading control. The bar plot reports the densitometric values of perilipin and PPARγ in the two mouse models normalized on the densitometric values of GAPDH (FAP-D2WT n = 4 and FAP-D2KO n = 5). E, Red Oil staining on GC muscle from FAP-D2WT and FAP-D2KO mice 7 days after glycerol injection. F, Hematoxylin and eosin staining on GC muscle from FAP-D2WT and FAP-D2KO mice 7 days after glycerol injection. On the right, cross-section area of TA muscle from FAP-D2WT and FAP-D2KO mice days after glycerol injection.

Following tamoxifen-induced D2-deletion, we injected glycerol into muscles of FAP-D2WT and FAP-D2KO mice, and collected the muscles 7 days later (Fig. 4A). We observed that FAPs isolated from FAP-D2KO mice exhibited lower mRNA levels of *Thra, Mct8*, and *Dio2* compared to those sorted from control mice (FAP-D2WT), whereas *Dio3* mRNA levels were increased (Fig. 4B). Interestingly, D2-depleted FAPs showed reduced adipogenic differentiation, as demonstrated by the decreased expression of adipogenic genes such as *Pparg*, *Pgc1a*, and *perilipin* (Fig. 4C). Consistently, FAP-D2KO muscle displayed a significant reduction in perilipin and PPARγ protein levels (Fig. 4D). Moreover, Oil Red O staining performed 7 days post glycerol injection confirmed a decreased number of Red Oil+ cells (differentiated adipocytes) in FAP-D2KO vs FAP-D2WT mice (Fig. 4E).

On induction of fatty infiltration, muscle homeostasis was determined by the balance between the activity of nonmuscle progenitor cells (like FAPs) vs MuSCs, which give rise to muscle fibers. We speculated that the impaired FAP activity would facilitate a myogenic program in the context of the FAP-D2KO muscle. To investigate this, we first assessed whether *Dio2* depletion in FAPs affects TH signaling–related genes in MuSCs. In uninjured muscle, the absence of D2 in FAPs did not alter the mRNA levels of *Dio2*, *Dio3*, or *Mct8* in MuSCs (Supplementary Fig. S2 (16)). However, *Thra* mRNA levels were slightly increased compared to control mice (*P* = .0017; see Supplementary Fig. S2 (16)). Following glycerol injection, no differences were observed in the mRNA levels of *Thra*, *Mct8*, and *Dio3* in MuSCs isolated from FAP-D2KO mice compared to controls. Interestingly, *Dio2* mRNA levels were decreased in this cell population relative to controls (see Supplementary Fig. S2 (16)). Next, we measured the myogenic process on glycerol-induced fatty infiltration in the presence or absence of D2 in FAPs. We analyzed the expression of myogenic transcription factors and myosin isoforms involved in myogenesis. In MuSCs isolated from FAP-D2KO mice 7 days after glycerol injection into the hindlimb muscle, we observed a decrease in early myogenic gene expression (*Pax7*, *MyoD*, *Myogenin*, *neoHMC*) compared to FAP-D2WT controls (Supplementary Fig. S3 (16)). Conversely, the expression of *Myh4* and *Myh1* (genes expressed by committed myoblasts) were increased, suggesting a more accelerated myogenic process (see Supplementary Fig. S3 (16)). Accordingly, the cross-sectional areas of fibers from FAP-D2KO mice were larger than those from FAP-D2WT mice (Fig. 4F). Thus, reduced adipogenesis due to the absence of D2 in FAP is associated with more effective muscle regeneration in the context of FAP-D2KO mice.

## Discussion

**Figure 5.**
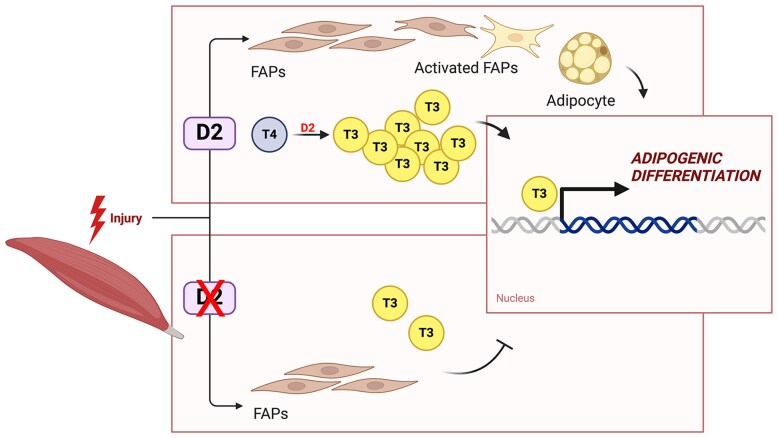
*Dio*2 from fibro-adipogenic progenitor cells (FAPs) decreases intramuscular fatty infiltration. Graphical illustration of type 2 deiodinase (D2) action in the transcriptional control of the adipogenic program in FAPs. This figure was created with BioRender.

In this study, we demonstrate that the regulation of intracellular TH levels by D2 is a newly identified factor in determining the cell fate of FAPs (Fig. 5). A cell-autonomous increase in D2 expression is essential for directing FAPs toward adipogenic differentiation. Enzymatic inhibition of D2 or genetic *Dio2*-ablation impair adipogenic differentiation and consequently reduce fatty infiltration of skeletal muscle.

The relationship between TH and cellular differentiation is a well-known concept. In the brain, TH are involved in neural stem cell fate. In particular, TH preferentially drives neural stem cells toward a neuronal fate (19-21), whereas a T3-free window, due to the presence of D3 and the absence of TRα1, is required for oligodendrocyte progenitor cell generation. Previously, we showed that in muscle, during myogenesis, D2 and D3 are expressed in MuSCs in a dynamic balance to tightly control intracellular T3 levels and thus modulate T3 concentration at each specific phase of the process (22, 23). In particular, we have shown that the proliferative phases of myogenesis require a hypothyroid status of myoblasts, which is achieved by enhanced D3 expression. On the other hand, myoblast differentiation requires an increase in the intracellular concentration of TH obtained by D2 expression. Intracellular T3, generated by D2, promotes *MyoD* gene expression and allows terminal differentiation. Similar to what has been observed in muscle, high levels of D3 activity and *Dio3* mRNA were found in proliferating brown preadipocytes, whereas D2 activity and mRNA levels are barely detectable in preadipocytes but are very high in differentiated brown adipocytes (2). These observations suggest that an intracellular hypothyroid state correlates with the proliferative state, while an increase in TH concentration is required during differentiation. Consistent with these observations, the results of the present work indicate that D2 expression is required to commit FAPs to adipocyte differentiation and to express their full adipogenic potential. Several pathological conditions such as sarcopenia, diabetes, cachexia, and muscular dystrophy are associated with intramuscular fat infiltration. Here, we have shown that the absence of D2 counteracts the development of intramuscular fatty infiltration, suggesting that controlling the expression of D2 could be considered as a potential tool to modulate adipogenesis in pathological conditions.

FAPs represent a unique population of mesenchymal stem/stromal cells found within skeletal muscle, playing a crucial role in muscle homeostasis, regeneration, and response to injury. They are characterized by their ability to differentiate into various cell types, including fibroblasts and adipocytes. In fact, FAPs are considered the main source of adipocytes during fatty infiltration of skeletal muscle (24-26). Several studies have identified signaling pathways involved in adipogenic differentiation of FAPs. Among them, Hedgehog signaling induces the tissue inhibitor of metalloproteinases 3, which in turn inhibits adipogenesis by inhibiting matrix metalloproteinase-14 (27). Moreover, to induce adipogenic differentiation of FAPs, WNT signaling must be switched off. Specifically, in FAPs, WNT5a reduces the levels of PPARγ, a master regulator of adipogenesis (28). We have previously shown that there is crosstalk between TH and both Wnt and Shh signaling. In particular, D3, the major inactivator of TH, is a direct transcriptional target of the β-catenin/TCF and of Shh/Gli2 complexes (29-31). We could speculate that in FAPs, when the Wnt and/or Hedgehog pathways are switched on, the expression of D3 is elevated and therefore the intracellular concentration of TH is attenuated, a condition that inhibits adipogenesis. On the other hand, the increase in D2 level that we observed when FAPs initiate adipocyte differentiation induces an increase in TH that could in turn attenuate Wnt-β-catenin and/or Hedgehog pathways (a mechanism we previously demonstrate to occur in colon cancer cells and keratinocytes, respectively (29, 30)). Here, we have shown that the rise in D2 expression during adipogenic differentiation of FAPs is associated with an upregulation of other genes critical for TH metabolism, such as *Mct8* and *Thra*. Accordingly, the TH-dependent signature is increased as indicated by the TRE3-TK-LUC activity. Interestingly, Fitzgerald et al (32) have recently identified a subpopulation of human and murine FAPs with pronounced adipogenic potential, characterized by the expression of the surface marker membrane metallo-endopeptidase. Taking advantage of the RNA-sequencing data published by Fitzgerald et al (32), we have analyzed the expression levels of *Dio2* in this FAP subpopulation and found that they express the highest levels of *Dio*2 compared to the other subsets and total bulk of FAPs (Supplementary Fig. S4 (16)). Similarly, increased adipocyte *DIO*2 expression has been found in subcutaneous and visceral adipose tissue from obese compared to lean humans (33). Based on the previous observations, we could speculate that *DIO*2 is a potential adipogenic marker, similar to *PPARG* (a bona fide TH-responsive gene (34)). This adds a new dimension to our understanding of the molecular mechanisms governing FAP differentiation and highlights the critical role of TH signaling in this process.

In summary, the possibility to modify intracellular TH concentration without altering circulating levels, thus avoiding the side effects associated with systemic hyperthyroidism/hypothyroidism, is a very appealing future scenario in clinical treatment. In this scenario, the recent approval of resmetirom, a selective THR-β agonist, for the treatment of nonalcoholic steatohepatitis is an example of how we can benefit from manipulating TH signaling specifically in some tissues without exposing patients to systemic dysthyroidism (35).

Our work could have potential applications by providing a better understanding of the role of TH in controlling FAP adipogenic differentiation. These findings suggest potential therapeutic applications for enhancing muscle regeneration through targeted modulation of TH signaling. In the future, further studies are needed to dissect the signaling pathway through which TH is able to regulate the adipogenic differentiation program.

## Data Availability

Original data generated and analyzed during this study are included in this published article or in the data repositories listed in “References.” Further information and requests for resources and reagents should be directed to and will be fulfilled by C.L. (cristina.luongo@unina.it).

## Abbreviations

cDNA: complementary DNA
D2: type 2 deiodinase
D2-KO: *Dio2* knockout mouse model
D3: type 3 deiodinase
*Dios*: deiodinases
FACS: fluorescence-activated cell sorter
FAPs: fibro-adipogenic progenitor cells
FBS: fetal bovine serum
GC: gastrocnemius
GFP: green fluorescent protein
mRNA: messenger RNA
MuSCs: muscle stem cells
qPCR: quantitative polymerase chain reaction
rT3: 3,3,5′-triiodothyronine
TA: tibialis anterior
T3: 3,5,3′-triiodothyronine
T4: thyroxine
TH: thyroid hormone
*Thra*: THR-α
THT: thyroid hormone transporter
TR: thyroid hormone receptor
WT: wild-type
